# Local Perceptions of COVID-19 in Pakistan’s Sindh Province: “Political Game”, Supernatural Test, or Western Conspiracy?

**DOI:** 10.1017/dmp.2021.220

**Published:** 2021-07-12

**Authors:** Inayat Ali, Salma Saddique, Shahbaz Ali

**Affiliations:** 1 Department of Social and Cultural Anthropology, University of Vienna, Austria; 2 Department of Community Health Sciences, Peoples University of Medical and Health Sciences for Women, Nawabshah, Sindh, Pakistan; 3 Independent Researcher, Islamabad, Pakistan

**Keywords:** local perception, COVID-19, pandemic, rituals, Sindh, Pakistan

## Abstract

**Objective:**

The coronavirus disease 2019 (COVID-19) has received various distinct perspectives and responses at the local as well as global levels. The current study pays attention to local perspectives, which have appeared in the Sindh Province of Pakistan.

**Methods:**

Given the constraints of the pandemic, and using convenience sampling, we conducted 10 online group discussions, 7 one-on-one interviews, and 30 cellphone discussions from a small town of Sindh Province. We made every effort to make our sampling inclusive in terms of decisive sociocultural factors: gender, religion, level of formal education, and occupation/job. We obtained data from women, men, Muslims and non-Muslims, the formally educated and noneducated, government employees, and daily wage laborers. Moreover, to perform content analysis, we used social media such as WhatsApp and Facebook.

**Results and Discussions:**

We have found that some people consider COVID-19 a “political” game, “supernatural test” or “Western plot”. The given perceptions then guide further actions: either ignore or adopt the preventive measures or take supernatural preventive measures. Considering it as a test of God, Muslims perform prayers, while the *Bāgrrī* community who practice Hinduism are taking cow urine to deal with the virus. This study brings these perspectives to the center stage; yet, the results cannot be generalized across the country, or within the province. Moreover, the study situates these perspectives within the global and socio-cultural, economic, and political contexts and invites more in-depth studies to inquire why such perspectives emerge.

**Conclusions:**

We discuss different narratives concerning COVID-19 in a small town of Sindh Province. We maintain that documenting these various perspectives and analyzing their impacts on the preparedness programs is essential, yet understanding the causes behind the stated standpoints is equally essential, if not more so.

In Pakistan, the Ministry of Health confirmed the first 2 coronavirus disease 2019 (COVID-19) infections on February 26, 2020: 1 person in Karachi and another in Islamabad. Within the next 15 days, the virus “officially” infected around 20 people, while the suspected number of infected people was around 470. The highest number of infections was in Sindh Province, followed by Gilgit-Baltistan.^1^ All confirmed cases had traveled from Iran, Syria, and London. Over time, infections rapidly escalated, and by the end of August 2020, the virus had infected around 30,000 people, of which over 6000 died. This spread led the government to impose “lockdown,” and then what the government called “smart” lockdown, which is the same as the “targeted” lockdowns imposed in the United States.^2^ Nevertheless, by the end of April 2021, the virus had infected around 826,000 people, of whom approximately 18,000 had died.

Owing to a rapid escalation of infection, the government re-imposed smart lockdown in 20 cities across the country, including the capital, Islamabad. By April 2021, as anticipated, what can be called the “third wave” of the pandemic caused a critical situation in Pakistan, despite the apparent efforts of the government to control the situation. These government efforts to contain the virus have included: early case detection and tracing and tracking contacts, communicating the risks of the virus to the public, physical distancing, quarantine, and isolation. Yet the novelty and scale of this disease, along with its uncertainty and ambiguity, have made it critical for health authorities to plan appropriate strategies and effective preparedness.

The continuous worries and fears have also created a fertile environment for local perspectives to emerge; the local perspectives of laypeople from villages and from a small town of Pakistan’s Sindh Province constitute the subject matter of this paper. We will analyze these perspectives after situating them within global as well as sociocultural, economic, and political perspectives.

## Methods

During May and June 2020, we carried out the study we report on here in a small town of the *Matiari* District located in the Sindh Province of Pakistan. Located in the southeastern portion of the country, Sindh is geographically 1 of the largest of the 4 provinces of Pakistan. We collected our data using well-established anthropological research methods. To conduct anthropological research, one needs ample time to build rapport, mingle with interlocutors, develop tools, and gather data through methods as well as observation. Although the research for this paper did not encompass long-term ethnographic fieldwork on COVID-19, it does draw on our earlier long-term ethnographic fieldwork projects in Pakistan, mainly in Sindh Province, which we have been conducting since 2005: Inayat Ali (2005 to the present), Salma Saddique (from 2013 on), and Shabazz Ali (from 2012 on). The fieldwork projects of Inayat Ali (2006 onwards, especially 2008-2011 and 2013-2020), and Shabazz Ali (2014-2020) on health and illness provide background and context for this present paper.

For the primary data collection in the village, we drew on Salma’s long-term fieldwork (2013-2020); for this particular project, Salma simply contacted her prior interlocutors. Given the constraints of the pandemic, and using convenience sampling, we conducted 10 online group discussions, 7 one-on-one interviews, and 30 cellphone discussions. We made every effort to make our sampling inclusive in terms of decisive sociocultural factors: gender, religion, level of formal education, and occupation/job. We obtained data from women, men, Muslims and non-Muslims, the formally educated and noneducated, government employees, and daily wage laborers. The purpose of this diverse sampling was to incorporate multiple and differing perspectives, as all these identities play pivotal roles in shaping the ideas and practices of the people in the selected locale.

Moreover, to perform content analysis, we used social media such as WhatsApp and Facebook. This study is part of a project that was approved by Pakistan’s National Bioethics Committee (reference No.4-87/NBC-471-COVID-19-09/20/); it included an informal interview guide. This guide encompassed 14 central themes related to COVID-19, including the necessary socio-economic information about our interlocutors on which this paper draws. The intention behind using an informal interview guide was to avoid affecting the views of interlocutors, as well as not making them feel uncomfortable with a “formal” interview. To maintain ethical considerations, we sought verbal consent from our interlocutors and have anonymized their names. Again, our analyses of our current data on COVID-19 build on our above-noted ethnographic fieldwork projects in Pakistan, including in Sindh Province.

### Different Perspectives on COVID-19 Around the Globe

While globally, (mis)information about COVID-19 is circulating rapidly around the sociocultural landscape, Pakistan has its own, unique socioculturally rooted (mis)information and (mis)conceptions about COVID-19.^3^ As the number of affected people increased, people’s perceptions of COVID-19 also gradually changed. Everyone is affected by the pandemic directly or indirectly, yet some people still consider it to be “propaganda.” After identifying over 2300 reports on media and social media in 25 languages in around 87 countries, Islam and colleagues^4^ have gathered the following 56 different local narratives: rumors, conspiracy theories, and narratives showing stigma (see Table 1).

**Table 1.** Rumor, stigma, and conspiracy theories related to COVID-19 around the world 2020

**Rumors** “Novel coronavirus is in the cloud” “Coronavirus is a snake ﬂu” “Pet animals are the sources of coronavirus” “Novel coronavirus strain is a type of rabies” “Coronavirus outbreak in the livestock” “Poultry eggs are contaminated with coronavirus” “Cookies, rice, and Chinese red bull were contaminated with the virus” “Eating bat soup is the source of the (COVID-19) outbreak” “COVID-19 found in oranges” “Coronavirus from imported goods” “ Mobile phone can transmit coronavirus” “Notes [currency] are sources of coronavirus” “Common cold had been renamed as coronavirus” **Rumor[s] about treatment, prevention, and control** “Eating garlic can cure coronavirus” “Drinking bleach may kill the virus” “Drinking alcohol may kill the virus” “Gargling vinegar and rose water or vinegar and salt may kill the virus in [the] throat” “Drinking cow urine and cow dung can cure coronavirus” “[Colloidal]Silver solution for coronavirus treatment” “Wearing warm socks, mustard patches, and spreading goose fat on one’s chest as treatment” “Keeping throat moist, avoid spicy food and taking vitamin C may prevent the disease” “Avoiding cold or preserved food and drinks, such as ice cream and milkshakes may prevent infection” “Spraying chlorine all over your body can prevent coronavirus infection” “Sesame oil can prevent coronavirus infection” “Granite bath can prevent coronavirus infection” “Sea lettuce can prevent coronavirus infection” “Vitamin C intake can prevent coronavirus infection” “Vitamin D can prevent coronavirus infection” “Eating Centella asiatica may prevent coronavirus infection” “Drinks containing mint or white willow, and spices like saffron, turmeric, and cinnamon would strengthen the lungs and the immune system against the virus” “Rinse mouths with salt water solution to prevent infection from the new virus outbreak” “Do not hold your thirst because once your membrane in your throat is dried, the virus will invade into your body within 10 minutes” “Applying petroleum jelly around your nostrils will protect against dangerous air pollutants” “Do-it-yourself coronavirus detection test” “Cannabis boosts immunity against the novel coronavirus” “Frequent washing clothes can reduce transmission” **Conspiracy theory[ies]** “Novel coronavirus is engineered, laboratory-generated virus either accidentally or deliberately released in the area of the Wuhan seafood and animal market” “COVID-2019 outbreak was planned” “It’s a bio-weapon funded by the Bill & Melinda Gates foundation to further vaccine sales” “Biological weapon manufactured by CIA” “President Donald Trump targeted the city with coronavirus to damage its culture and honor in Iran” “The virus is an attempt to wage ‘economic war on China’” “America’s Jews are driving America’s wars” “This outbreak is medical terrorism” “Zionists are against regional security” “United States and Israel of being behind the creation and spread of the deadly coronaviruses, [which are] part of an economic and psychological war against China “This outbreak is a population control scheme” “Tom Cotton claimed that COVID-19 was manufactured in [a] Chinese bio-laboratory” “Rush Limbaugh opining that whole COVID-19 is a conspiracy against Trump to let him down in election. He purported it as a worse ﬂu” “New coronavirus vaccines already exist” “Pneumonia vaccines are effective against the Wuhan coronavirus” “Israel has sent a vaccine to Wuhan city for patients infected with coronavirus” **Stigma** “I am not a virus: French Asians angered by racism” “Chinese are uncivilized” “Chinese are bioterrorists” “A French newspaper with headline ‘Yellow Alert’ tagged an Asian woman image wearing mask” “Chinese are dropping their coronavirus” “Every disease [ever] has come from China” “Keep your virus, dirty Chinese” “Chinese dietary habit[s] caused COVID-19”

### COVID-19 From a Local Perspective in Sindh

While all of these perspectives are fascinating, herein we focus on Pakistan, as, although Islam and colleagues have paid significant attention to these varying narratives, they have not included anything from Pakistan. As in other countries, various local perspectives and narratives about COVID-19 have emerged in that country.^3,5–7^ For a non-COVID-related example of how rumors and conspiracy theories can spread in local areas, Inayat Ali reports that he repeatedly heard varying narratives in Sindh Province concerning an unfamiliar, aggressive, and dangerous small animal that appeared and attacked in 2016. This story held that:

the animal was an Indian conspiracy to cause havoc in Pakistan—someone imported them from an Indian zoo to deliberately set them free in Pakistan. The other held that the animal had emerged from an unknown area. As fear grew larger and stronger, villages started assigning duties to various individuals to provide 24-h security. People, especially children, were afraid to sleep. The male members stopped sleeping at an Otāq (a male guest house) or walking outside the house after sunset. Frequent announcements broke out about seeing the animal, which caused villagers to gather with their dogs and their weapons—rifles, pistols, axes, and sticks. These gatherings attracted the local media. A few deaths occurred, in which the bodies were said to contain “paw”-like marks, which added further fear into an already chaotic situation. Despite the fact that such an animal was never captured, the rumor continued unabated for over a month.^8^

Similarly, multiple competing narratives have been surrounding vaccination campaigns, that is, that these campaigns contain “hidden interests” and are a “Western plot” to sterilize Muslim women, leading to vaccine refusals and resentment.^8^ The “Western plot” and “hidden interest” narratives became a reality when the media reported a fake vaccination drive that the American Central Intelligence Agency (CIA) organized in Pakistan’s Abbottabad city to locate Osama bin Ladin. This critical event was termed “vaccination suicide” as it significantly affected the Expanded Programme on Immunization (EPI); many vaccinators were attacked, and over a hundred were killed.^8^

Likewise, various narratives surrounded COVID-19 to locate the “hidden agent” behind it. Some believed that either the United States or Big Pharma has “bioengineered” COVID-19. People also started circulating information about home remedies, eg, drinking garlic water (which might actually help), or “blowing hot air from a hair dryer through your nostrils”; or keeping one’s throat moist on the recommendations of the country’s health ministry.^9^ Two highly prevalent narratives in Sindh Province were about treatment:

1. A widespread rumor broke out in Sindh Province, from its district *Hyderabad* to district *Ddaharki*, that shaving one’s head protects against the virus. As soon as this rumor traveled, many men (the shaving of a woman’s head is considered shameful) immediately shaved their heads—a very affordable preventive measure that costs only US5-10 cents. In 1 village of Sindh, over 50 men had their heads shaved. This rumor is a good example of how a society can come up with an easily accessible “cure” in the absence of a practical and effective health-care system.^5,6^

The second narrative emerged concerning an infant in the northern part of Sindh Province, which quickly spread in many districts with multiple forms but with the same idea as given below:

1. After this baby’s miraculous birth, he started talking: “I will not survive. I am here to tell you something important about the current coronavirus, that the disease is deadly. I will die at noon and will bring the coronavirus with me,” said the child. It could kill everyone if a recommended measure was not taken. The measure was brewing green tea, and every person should drink 5 sips. The one who would drink these 5 sips would survive; the rest would die. “As long as my heart beats, I ask you to please drink tea.” After conveying this message, the child died.^5,6^

Following the wide dissemination of this narrative, many laypeople got on their mobile phones to make calls and send messages to their family, friends, and acquaintances. Some even paid in-person visits to their neighbors to bring the great news of this miraculous treatment, showing the willingness of the locals to believe in the supernatural.

In contrast, some local people believe that COVID-19 does not exist but is just a “political game” being played because the Pakistani government wants funds from high-income countries and from the International Monetary Fund (IMF). Inayat Ali discovered that many people in Sindh and Punjab Provinces believe that “the government is only imposing lockdowns to receive global attention for potential foreign aid and that there is no danger.”^5,6^ Therefore, many believe that the government is “fabricating” the statistics, which is why the number of infected people is escalating. Some interlocutors believe that COVID-19 is a *Yahudi Sazish* (Jewish conspiracy) to stop Muslims from going to mosques. Those who do not believe that this pandemic exists freely go to markets and roam around. They believe that there is no reality in the coronavirus as presented in the media; they reason, “If COVID-19 exists, why we have not been affected?”

In contrast, some Pakistanis who do believe that it is real fear contracting it; hence, they have been maintaining self-quarantine. Sorath—a 35-y-old woman from Sindh Province working in a nongovernmental organization (NGO) in *Matiari* District —stated that:

Our life is totally at risk. We are fearful of contracting the virus, as the pandemic has affected millions of people worldwide. Owing to that fear, there are people who even have a simple cough; they think they have contracted the virus. My uncle died due to heart failure. Since he had symptoms of COVID-19, he was brought for a test for this virus to a nearby hospital. On his way, he got heart failure because he was significantly under stress and fearful of contracting COVID-19. Consequently, he died when he was on the way to a hospital. He was worried, like many people in Sindh, to be tested for the coronavirus due to the attached fear that he will be put in quarantine.

Moreover, some interlocutors perceived that the coronavirus is only dangerous when one does not follow the required measures, such as physical distancing, wearing masks, and using a sanitizer. If a person follows precautions, eats a healthy diet, and does not go outside, then s/he is safe from this virus. As Qudarat, who is in her late 40s and a gynecologist at a government hospital of *Matiari* District, explained:

COVID-19 has created an alarming situation for us. We must take precautions because we will also be dealing with it soon. It is better to make our body prepared and strong while eating a healthy diet and practicing physical distancing. The most important thing is to make our minds strong enough to deal with this critical disease.

Most interlocutors argued that they have not in actuality met anyone infected with COVID-19; however, they have constantly been reading and watching about them on social and print media. Thinking about COVID-19 has resulted in psychological depression in some people, as they feared that the government would enforce quarantine and isolate them from their families. And being diagnosed with COVID-19 may stigmatize them in the eyes of others. Habiba, a 29-y-old from the Matiari District, who also works in the private sector, shared that:

One of our colleagues has tested positive for COVID-19; therefore, our organization has decided to conduct COVID-19 tests of all employees and communities who remained in physical contact with the employee in the last week. Nonetheless, due to fear, people are refusing to get the coronavirus tests. The underlying reason is that if they are tested positive, it will mean shame for the community and the government will not allow them to live with the family members, and we will be put in an isolation center. Yet some people consider it an opportunity: if tested positive, then they will be spared from work.

As Habiba notes, for some people COVID-19 has emerged as an opportunity to get some free time to spend with their families; a colleague of Habiba, named Halima, who is in her late 30s from Matiari District, stated, “We want our COVID test to be positive because after declaring as positive, we will take a rest for a few days, as our organization will give us leave without pay. And, this way we will have time to spend time with family.”

One year after we obtained our original data for this paper, the current situation of COVID-19 became more critical in Pakistan as people got used to the virus and become less or nonserious about protecting themselves from it. This criticality was hinted at almost a year ago by our interlocutor Sakeena, who is around 40 y old and works as a nurse at a private hospital in a town of Matiari District, was of the view that:

When the coronavirus infection was first confirmed in Pakistan, most people were following precautionary measures, such as wearing masks and gloves, observing physical distance during going outside from home. However, the current condition is critical because many people are not taking COVID-19 seriously anymore. People have become habituated to it by now.

Some people also have related the etiology of the pandemic to a supernatural act. Inayat Ali^5,6^ found interpretations of COVID-19 as a supernatural act in Punjab Province, where laypeople considered the virus as the punishment of Allah, which occurred due to “the opening of cinemas in Saudi Arabia and general disbelief in God in the Global North, where everything is ‘too open,’ especially romance.” People believed that “Allah has shown us how powerful He is in that science is unable to deal with the pandemic.” Many Pakistanis hold the idea that their strong belief in Allah is the reason why the virus infections are significantly less in Pakistan than in the United States and Europe.^5,6^ For example, Asad Ali, age 45, who is a college teacher from Matiari District, argues:

COVID-19 is a punishment for unbelievers [this may be translated: those who do not practice Islam]. As we are followers of Ali (the son-in-law and cousin of the prophet Muhammad), we would not be infected by the virus under any circumstances. [In Urdu] *Jis Ka Ali Waris, Usay Kiya Kre Ga Coronavirus* [literally meaning: If Ali is our protector, then we won’t be affected by the coronavirus].

Similar are the views of Kamalan, who is around 60 y old from the same District with no formal education and a house worker, who stated:

None of us has a fear of coronavirus; instead, we are afraid of God. The occurrence of a disease and its cure is the will of Allah. Nobody except Allah would ever cause any disease to anyone. Despite that, we are living in the scientific world and cannot make or unmake an illness because it occurs by the decision of Allah. Presently, the coronavirus has been described by a religious leader and many people as a disease from Allah to test individuals and put them on the right track. Many people are stating that the coronavirus spread because people are not performing *Namāz* (praying) and [reading the] *Qurān*. Once Allah is pleased with us, then the disease will soon end, as we are not able to prevent ourselves from the disease—only Allah can do so.

Of interest, the Bāgarrī—a nomadic Hindu hunting and gathering community of Sindh—have adopted a distinct set of preventive measures. They have performed various rituals and have used cow urine for prevention because they consider cows to represent a goddess—which particular goddess depends on the specific Bāgarrī cultural group, as each group worships a different goddess. As Jadul, a 50-y-old Bāgarrī woman with no formal education who works as a wage laborer, shared:

We used to wash our bodies with cow urine as the cow *Mātta* [a term that is used for Hindu goddess by the practitioners of Hinduism] is our goddess. Firstly, we made sweet bread and distributed it to children, and then the children offered the bread to a cow. After an hour, when the cow urinated, then we kept that urine in a metal jug and recited some *Bhajjan* [sacred song of Hinduism]. After that, every family member, irrespective of age and gender, washed their body with that urine. Furthermore, we also sprinkle urine on every space of our house. Our deliberation is that the virus will not affect us, with the blessing of our cow Mātta.

Other cultural groups have also used cow urine while considering it a sacred measure to deal with COVID-19. Holding a similar belief, members of All India Hindu Mahasabha (All India Hindu Union) organized a *Gāūmūtrā* (cow urine worship) program, hosted by *Akhil Bharat Hindu Mahasabha*. Hundreds of people attended this event and drank the urine, believing that this would immunize them against the virus. (A specific type of cow urine is used in Ayurvedic medicine, in which it is believed to have antioxidant and anti-microbial properties.)

## Discussion

People’s beliefs and perspectives are real, influence behavior, and help them to make sense of their lifeworlds.^3,5,10,11^ These beliefs and perspectives are encoded in various narratives, eg, rumors and conspiracy theories, which are social phenomena that reflect people’s anxieties and fears.^5^

Believing that there is no coronavirus reveals a contestation between different medical systems, and also demonstrates the mistrustful relationship between the government and the people, which, again, creates negative effects as people have become more careless in taking preventive measures against a disease they do not believe even exists. The deeper meanings of such perceptions can only be fully understood when they are placed within the historical context of colonization, poverty, corrupt or ineffective governance, and aid dependency.^8^

Some narratives can be downright dangerous. For example, based on a rumor that consuming highly concentrated alcohol disinfects the body and kills COVID-19,^12^ people started drinking alcohol excessively, and as a result, around 800 people died in Pakistan, and around 5900 in Iran.^13,14^ Some Americans died from drinking bleach because then-President Trump actually recommended it, and Asian Americans across the United States are being vilified, attacked, and even killed because to some, they incarnate the belief that the virus was deliberately imported to the United States from China. Former President Trump repeatedly referred to COVID-19 as the “China virus,” which apparently strengthened the association of Asians in general with the coronavirus in the minds of his followers, expressed through social-media hate speech and now actual attacks.^15^

Rumors, conspiracy theories, and infection-associated stigmas may also negatively affect people’s willingness to comply with government preparedness programs to contain the virus.^5^ Such narratives may also exert positive effects. For example, linking the pandemic to the supernatural likely gave laypeople great hope that pleasing and worshiping Allah would help to prevent and cure the disease. For another example, Inayat Ali^3,5^ found that, at the beginning of the pandemic, rumors circulated in Pakistan that the government shot or set fire to COVID-19 infected people. These narratives made family members, particularly parents, to be highly concerned about the mobility of the younger generation so that they should stay at home.^3,5^ On the upside, these rumors might have caused people to intensify their efforts to avoid contagion by self-quarantining. Yet on the downside, such rumors caused many to avoid getting tested even if they were symptomatic.

Thus, such local perspectives may both create panic and decrease fear. For example, believing that health and illness, including COVID-19, are determined by God allows people to release their anxieties and fears through prayers. Similarly, listening to the sacred songs of *Bhajjan* (Hindu religious songs) also gives hope to people that the virus will not infect them. According to Davis-Floyd and Laughlin, “ritual stands as a buffer between cognition and chaos”—between people’s ability and inability to cope with a stressful situation—because the performance of rituals can be calming and stabilizing.^16^ Thus, it is no wonder that people everywhere use rituals to stabilize themselves in times of crisis.^16^ Even the small daily rituals of handwashing, mask-wearing, and physical distancing give people a sense of confidence, while deeper rituals, such as performing prayers and large-scale ceremonies like the one described above that involves drinking cow urine, greatly enhance that sense of confidence.

Knowledge, attitudes, and practices toward any disease play an integral role in shaping society’s readiness to accept behavioral changes. The lessons learned from the severe acute respiratory syndrome (SARS) outbreak are that knowledge and attitudes are associated with levels of panic and emotion, which further complicate measures to contain the spread of the disease.^17^

These perceptions and practices are shaped by sociocultural, economic, and (geo-)political factors. Remaining distinct from society to society, they significantly impact an individual’s life, as is widely understood. Cross-cultural studies support that each specific culture has its own beliefs related to particular explanations for health and sickness.^18^ When facing new and challenging diseases causing epidemics and pandemics, we should consider these different perceptions and practices because they may affect how symptoms are recognized, access to care, treatment provided, and fear of stigmatization. Public health interventions should consider cultural beliefs and assumptions to ensure that the interventions are culturally appropriate for the community.^19,20^ It is crucial to avoid correlating the disease with questionable cultural causations, as this may lead to blaming specific populations for their high prevalence rate or to the stigmatizing of those infected.^21^

We note that it is essential to conduct in-depth studies to generate a thorough understanding of these numerous perspectives: what do such narratives reveal and why do they emerge? Documenting them, their significant impacts, and their underlying causes, as we have done herein, is undoubtedly vital. Such documentation and analysis will lead us to those historical, sociocultural, economic, and political factors that significantly shape these seemingly irrational perspectives, which, in Pakistan and many other countries, develop in the presence of multiple and syndemic disparities, including lack of effective educational and health-care systems and to an inefficient and corrupt governmental regime.^5,6^

## Strengths and Limitations of the Study

Because the perspectives surrounding COVID-19 are still developing, this study would be among those limited investigations that have focused on the local perspectives and practices on the disease in Pakistan. Moreover, analogous to other qualitative studies, this study has certain limitations. The sampling has certain limitations: (a) its type, that is, convenience sampling, (b) the number of interlocutors is small, (c) most of the interlocutors are women. Therefore, the results cannot be generalized to the entire country. Yet our study offers first-hand data and triggers further studies following the same lines in Pakistan. Moreover, it adds a perspective from Pakistan to other studies that have been documenting and analyzing different narratives around COVID-19 at a global level.

## Conclusions

Analogous to other countries, Pakistan has been overwhelmed by the pandemic. Because COVID-19 is still unfolding and there appears as yet (May 2021) no or not enough vaccines available in many countries, including Pakistan, and distinct perceptions, attitudes, and practices surrounding COVID-19 have emerged there, as elsewhere. We have described these in this paper, showing that the practices around COVID-19 in Pakistan range from harmless home remedies like drinking green tea to extremely harmful “preventive measures” like drinking oneself to death. We have also shown how local beliefs and attitudes about COVID-19 have strongly influenced behaviors in both positive and negative, rational and irrational, ways.

Understanding local attitudes, beliefs, and practices and their underlying rationales could greatly help health officials to present science-based strategies for preventing infection in culturally appropriate and acceptable ways. For example, government protocols could recommend drinking green tea and performing rituals and prayers, harmless practices that carry great psychological benefits, while noting that the “beneficial” effects of such home remedies or large-scale ceremonies would be greatly enhanced by also wearing masks, hand-sanitizing, and physical distancing. In such ways, government standard operating procedures could be presented as a package that includes local remedies and honors local beliefs. This present research on Sindhis’ perceptions of COVID-19 can greatly aid in such efforts.
